# Supplementary material for: SPL12 Regulates AGL6 and AGL21 to Modulate Nodulation and Root Regeneration under Osmotic Stress and Nitrate Sufficiency Conditions in Medicago sativa
Source: Plants (Basel). 2022 Nov 13;11(22):3071. doi: 10.3390/plants11223071 (PMC9697194; doi:10.3390/plants11223071)
Supplement: Supplementary file 1 [file plants-11-03071-s001.zip › plants-1951856-supplementary.pdf]

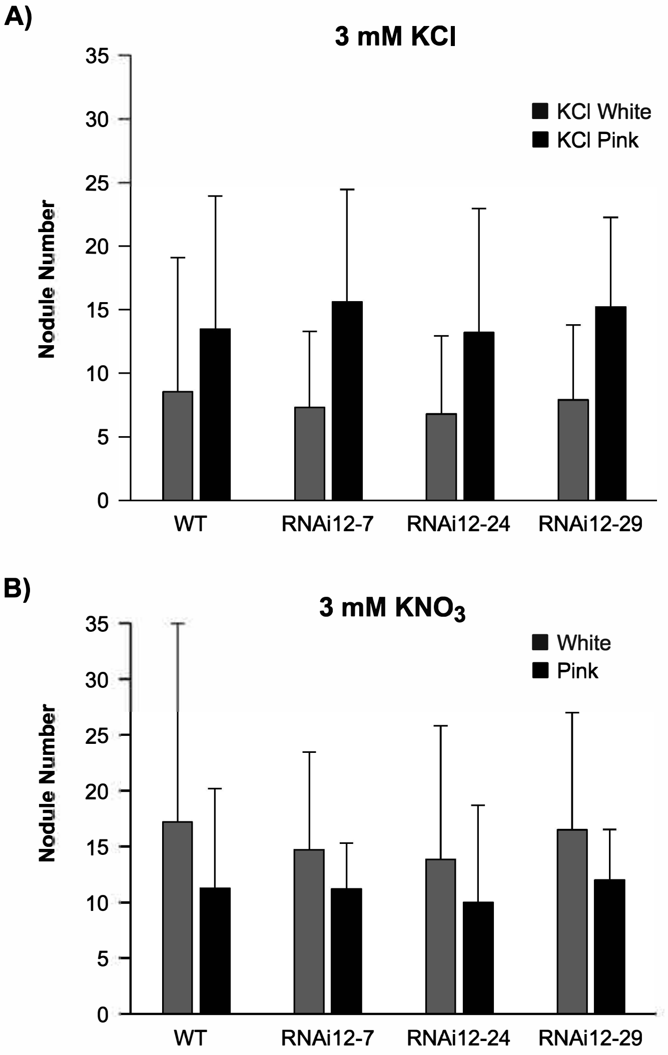

Figure S1. Effect of nitrate on nodulation in *SPL12*-RNAi plants.
The average numbers of pink and white nodules in WT and *SPL12*-RNAi at 21 dai (n = 15-22) under 3 mM (a) KCl and (b) KNO_3. Error bars indicate standard deviation.

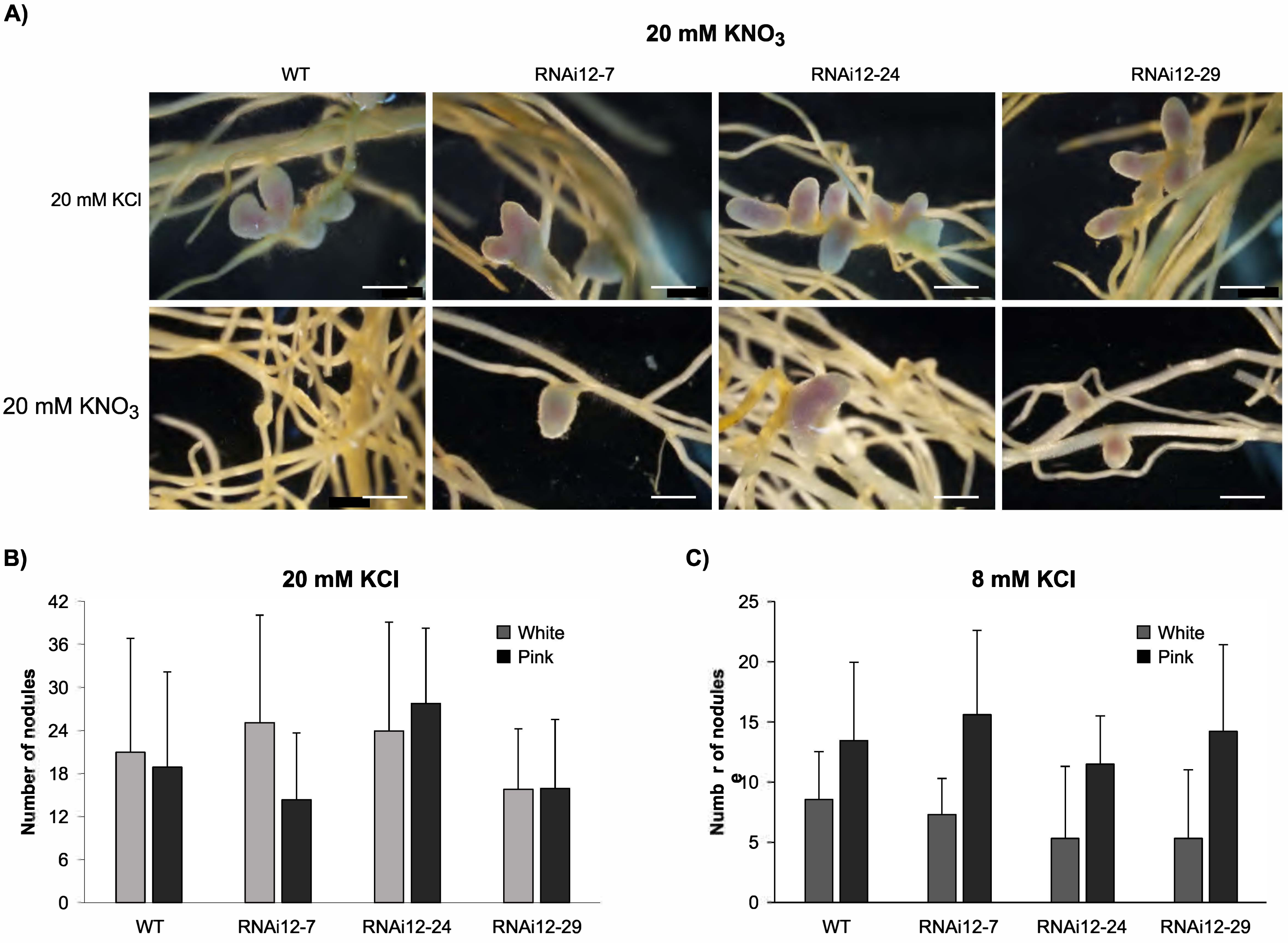

Figure S2. Effect of nitrate on nodule phenotype in *SPL12*-RNAi plants.
(A) Nodule phenotypes of WT and the *SPL12*-RNAi genotypes at 21 dai growing in nitrate-starved substrate and watered with 20 mM KCl or KNO_3. Scale bars: 1 mm. The average numbers of pink and white nodules in WT and the *SPL12*-RNAi at 21 dai under (B) 20 mM KCl (n = 14-25 plants) and (C) 8 mM KCl (n = 15-22). Error bar indicates standard deviation.

GGAGGTTACAACTATATATACTAATAGGACTAATACTACTTGTAACTAAACTATGTATCTCTAATAGGTGTTTTGATGTGCGAGGTGTAAGGTT
TTCTTTTGGTGTTCCGCATATATACGACGTCTTTTGTTATGTTGTTTATTCTTACCGGTCTCTAAAACATTACTCTTTAAAAAAATATGAAAGAT
TTTTCAATTTCTCCTATATTTCTCACCCCTAATGTGTTCACCTAAACCTCTAAGATTCTAAAACATGAACTTTTTCTTTTTGGAGATACCCTTTT
TAGAACGGGTGATTATGCCTCCTGAATTTGTATCAGCAGGAGTGACAACATCACTTTGCTTCTTTTTTTTTCTGCAGTTCCATGTTAACCTTT
GTTAGAGTTTCCACATGTTTGTTGGGATTGTATTCCTGCACAATTAAAGTGTTTGCGTCAGGATCATCCTCTGCTAAGTCACCCCAATGTTCC
TTAAGGAT[illegible]GTACACTCCTCTTACTAGCCTCTGTGGGTACACTCCTCTTACAAGCCTCATCAAATTTAACAAAGTTTTTCTTCTCAAACAA
AAAAACTCTTATCCCTTTAAGCTTCTAGCTCCACTCACCCATTTTTCGAATGTAGATCTCTTCCTACTCGGATATTGCCAACAACCTAAGGAA
AACTTGCTTAAGAGAGTAGACATCAATACATCTATCATGTCAAAATTGAATTCTCCCTTCATCTTCCATTTTTTTCACACGCTAAGCTCGTAAAT
TGATCCCCTTTAACCCTTCACATACGGTCTAAACAACACAAATTGTTCCATCACAAAGGAAGTTGATGACTCTGGAGAAGCCAAAGTGCTA
ACACCATACTTGGTTGACAAGACGTTTATCCATTGAGACTACCTTTATTAATAACTGCCACCTCCATTTGGTTGAAATATATTGTCTACACATG
TCCCCTAATGAATAAAGTATCAGTACGCACGCTCCCAGAAAGCGTGCGAGTGAGGACAAAACACGCTGAAAAGATTTGCGTTGATTTGTG
GGGTCAGTCGGCAATCCTAAAAGCAGTCTGAGATGTTACATTGGGAGCCCTTCTGGAGGGCACCTAAGG CCTCTCTAGAAGGCATTTGTTC
CCTTAGGAATAGATTGACGATTCAATTATATTATTAAGTTTGTGTAAAATGACCAAAATGGTTTATGAGA AAGTACGTCTTGTGCAAAAATAC
TTTTTTTAAGAGGCTAGACGTGTTATCGTCATGCCTTATATTGATAAGGCTTTTAATTTTTATATTCTTTTAGAATGGCAAATGTTATTAATGAA
TTGTTAGAATAATTTCTCTCCTTTATCCGAGTTTAAACTCAGGAACTTCAACTCCTTTATCTCTTGGCTCAAACCAGTTGAGTCATCCAACCTC
CATAGATTAAGCATTTCATGATCAATGATATTGATAATTTTGGGAACAAATTAAAAATATGATTATTCTTTCTTGTGGTCAGC ATGAAATCAGT
GATTGAGAGATATAACATTTGCAAGGAAGACCAACAAGGGACAAATCCAGAATCTGAAGTCAAG

Figure S3. Promoter sequence of the alfalfa *AGL21* gene with putative SBD binding elements. Nucleotides highlighted with green/yellow represent putative SPL binding elements with 'GTAC' core sequences, those highlighted in blue represent forward and reverse primer sequences used for ChIP-qPCR. The red text shows coding sequences of *AGL21*.

Table S1: List of primers used in this study

Primer name	Primer Seq
LA-MsSPL12-F1	CCCCCAAACCAAAGATTTTA
LA-MsSPL12-R1	TCTTGGTTCCTTTGCCTTTG
qMsAGL6-1F	TGTTATGTGATGCTGAGGTTGC
qMsAGL6-1R	GTTCTCCTAAGTCACCATTCTCAG
AGL21-F	GGGAGGCAGAAATTTTAAGG
AGL21-R	TGGAGGCTAAGTTCCAGTTGA
β-actin-F	CAAAAGATGGCAGATGCTGAGGAT
β-actin-R	CATGACACCAGTATGACGAGGTCG
ADF1qF	TCAAGGCGAAAAGGACACAC
ADF1qR	AAAACAGCATAGCGGCACTC
CycloqF	CAAACTTTCCTGACGAGTCACC
CycloqR	ACGGTCAGCAATTGCCATTG
qMsLOBF	AGATGGTGATTCGTGACCCG
qMsLOBR	CTGTTGCTGCTGTTGTTGATTC
LysM-F	TTGGAGTCGTGCTGTTGGAA
LysM-R	AGCTTTCGATGAGGCAAGGA
CLE13-F	TCCTCACACCAAGAGTTTATGCT
CLE13-R	TCCTGTAGGCACTTTGCGTT
SULTR3.4-F	TGGAAATTCACAGGGTTCGT
SULTR3.4-R	CTCAGGACCCCATTGAAAAA
SULTR3.5-F	GCACTTTTCCCTGATGATCC
SULTR3.5-R	TGGCAAGACTAGCAATGGTG
q1MsAGL21-F	AAGTGTTTGCGTCAGGATCA
q1MsAGL21-R	CGAAAAATGGGTGAGTGGAG
q2MsAGL21-F	CCACCTCCATTTGGTTGAAA
q2MsAGL21-R	TCAGCGTGTTTTGTCCTCAC
q3MsAGL21-F	CCTCTCTAGAAGGCATTTGTTC
q3MsAGL621-R	AAGGCATGACGATAACACGTC
GSH-F	ACGCTTCCCAGCTTTAATGA
GSH-R	CCCCAACAAGAAGACCATTG
WD40-1-F	GGATGAATCTGTGAACGCCG
WD40-1-R	CTTTGTCCACGGCTCAAACA
